# The Physician’s Opportunity

**Published:** 1916-06

**Authors:** 


### EDITORIAL DEPARTMENT

MRS. F. E. DANIEL, Publisher and Managing Editor.

### The Physician's Opportunity.

Surprisingly few physicians become wealthy. It is because most of them become more interested in their studies and their practice than in their business. Then, in the nature of things, those who are not deeply interested in the professional side of their work soon have but little business to which to give attention. It is rather an anomalous situation; those who are progressive as professional men are inclined to neglect business, while those who are not progressive inevitably lose their business.

The result is that many physicians come in time to regard themselves as public benefactors, and are satisfied to devote much of their time to the public without any expectation of reward. Medicine is the most unselfish of all the professions. The man who goes into it with the thought of self-aggrandizement either in wealth or in reputation is likely to be disappointed. Where one secures these hundreds remain in poverty and obscurity.

But neither poverty nor obscurity can keep a man from being useful, and, after all, useful service is the best test of a person's real character. The world is learning that the best purpose with which to fill one's life is the desire to do genuine service to others,—to live so that when life is ended one can have the feeling that he has contributed something to alleviate suffering and to bring joy into the lives of others. Measured by this standard, what wonderful privileges are accorded every physician? There is not a day that the active physician has not some opportunity to make some human being happy. He can carry happiness into the humblest cottage and the richest mansion alike, and can do it without any loss to his professional dignity or standing. His is a remarkably close relation to his patrons, one that gives him excellent opportunity to do good. If he improves that opportunity, he may well feel that his life is usefully spent, regardless of whether he achieves either fortune or fame.

